# The Hand Book of Human Anatomy, General, Special, and Topographical

**Published:** 1847-08

**Authors:** 


ARTICLE X.

*The Hand-Book of Human Anatomy, General, Special, and Topographical.* Translated from the original German of Dr. Alfred Von Behr, and adapted to the use of the English Student. By JOHN BIRKETT, Fellow of the Royal College of Surgeons of England, and Demonstrator of Anatomy at Gray's Hospital. Philadelphia: Lindsay & Blakiston. 1847. pp. 487. (From the Publishers.)

The small anatomical work with the above title, gives a short and comprehensive detail of anatomical facts, and shows the relations which Anatomy bears to Physiology, Pathology, and Surgery. It is well adapted to serve either as an introduction to anatomy, or for refreshing the memory of practitioners, or students in the intervals between courses of lectures. H.

